# Influence of Tree Species Composition and Community Structure on Carbon Density in a Subtropical Forest

**DOI:** 10.1371/journal.pone.0136984

**Published:** 2015-08-28

**Authors:** Yanqiu Hu, Zhiyao Su, Wenbin Li, Jingpeng Li, Xiandong Ke

**Affiliations:** College of Forestry, South China Agricultural University, Guangzhou, Guangdong Province, China; Wuhan Botanical Garden, Chinese Academy of Sciences, Wuhan, China, CHINA

## Abstract

We assessed the impact of species composition and stand structure on the spatial variation of forest carbon density using data collected from a 4-ha plot in a subtropical forest in southern China. We found that 1) forest biomass carbon density significantly differed among communities, reflecting a significant effect of community structure and species composition on carbon accumulation; 2) soil organic carbon density increased whereas stand biomass carbon density decreased across communities, indicating that different mechanisms might account for the accumulation of stand biomass carbon and soil organic carbon in the subtropical forest; and 3) a small number of tree individuals of the medium- and large-diameter class contributed predominantly to biomass carbon accumulation in the community, whereas a large number of seedlings and saplings were responsible for a small proportion of the total forest carbon stock. These findings demonstrate that both biomass carbon and soil carbon density in the subtropical forest are sensitive to species composition and community structure, and that heterogeneity in species composition and stand structure should be taken into account to ensure accurate forest carbon accounting.

## Introduction

Forests play an important role in maintaining the carbon balance between terrestrial ecosystems and the atmosphere [1]. In the Kyoto Protocol, forest carbon sinks have been acknowledged as effective channels for offsetting greenhouse gas emissions [2, 3]. Under the Clean Development Mechanism (CDM) and emissions trading, worldwide afforestation and reforestation projects for carbon sequestration have been initiated, thus advancing related work on the measurement, monitoring, and evaluation of carbon stocks and sequestration potentials in forest ecosystems [4, 5]. However, carbon accounting methodologies for CDM-based forestry projects are oriented towards large-scale applications, and the results are often highly variable and uncertain [6, 7]. In addition, the measurement of carbon stocks in non-CDM based forests, especially natural forests, is largely dependent on the accounting protocols used for CDM-based afforestation and reforestation projects [8–10]. Consequently, spatial heterogeneity in forest communities and differences in community structure have not been adequately reflected in these methodologies.

Given the growing importance of forest carbon trading in the post-Kyoto era [11, 12] and the increasing need to assess forest ecosystem services [13, 14], it is necessary to refine the methodologies used to measure forest carbon stocks. To advance stand-level forest carbon accounting research, researchers should examine the impact of species composition, community structure, and age, as well as the spatial distribution patterns of carbon stocks within forests. It is thus crucial to increase the precision of the measurement of carbon stocks and to improve the methods used to assess ecosystem services to sustain the forestry industry.

Our study focused on a subtropical evergreen forest. We examined the impact of species composition and stand structure on forest carbon density and investigated the relationship between forest biomass carbon density and soil organic carbon density. The purpose of our study was to determine how forest biomass carbon and topsoil carbon densities or stocks change across spatial and species-specific compositional and size gradients. Understanding these relationships will improve the measurement of forest carbon stock and help guide species selection in afforestation and reforestation projects used for carbon sequestration in the subtropical region.

## Materials and Methods

### Study area

The study area was located in the Kanghe Provincial Nature Reserve in Dongyuan County, Guangdong Province, China (115°04′–115°09′E, 23°44′–23°53′N). Before we set up sampling plots for plant inventory and soil sampling, we obtained permission from the Kanghe Provincial Nature Reserve Administration Office. The area of the nature reserve is hilly, and the mountains run in a northeast to southwest direction, with the highest peak at 839.7 m a.s.l. The area has a subtropical monsoon climate with abundant sunshine, a mean annual temperature ranging from 20.3–21.1°C, and a frost-free period of 345–350 d. The average annual precipitation is 2,142.6 mm, and the annual mean relative humidity is 77.0%. The soil is a clay loamy latosolic red soil and includes a thick soil layer. Vegetation is of the subtropical broadleaved forest type, dominated by the evergreen tree species *Castanopsis carlesii*, *Schima superba*, *Castanopsis fargesii*, and *Itea chinensis*. In a few locations, coniferous trees such as *Cunninghamia lanceolata* and *Pinus massoniana* are abundant in small patches.

### Plant census

A 4-ha permanent sampling plot was set up within the nature reserve for the survey of the evergreen forest. The plot was further divided into 100 contiguous 20 × 20-m quadrats using a total station (Nikon DTM-310). The elevation of the sample plot ranged from 216 to 345 m a.s.l., and the slope ranged from 15.3° to 45.7°.

All individual trees ≥ 1 cm diameter at breast height (DBH) were tallied, tagged, and recorded by species name, DBH, and tree height. Standing trees ≤ 6 cm DBH were measured using a dial caliper, whereas trees > 6 cm DBH were measured using a diameter tape. Tree height was measured using a measuring rod for trees ≤ 6.5 m and using a clinometer (Suunto PM-5/1520, Finland) for trees > 6.5 m. DBH was measured to the nearest 0.1 cm, and tree height was measured to the nearest 0.1 m.

### Soil sampling and determination

Bulk samples of topsoil (0–25 cm depth) were collected at the center of each 20 × 20-m quadrat using a stainless steel core cutter to determine soil bulk density. To determine soil organic carbon content, subsamples of topsoil were first collected at the one-quarter, center, and three-quarter points along a diagonal line within each quadrat; these subsamples were evenly mixed to obtain a 1-kg composite soil sample per quadrat. The composite soil samples were subjected to potassium dichromate oxidation to determine the soil organic content. Soil organic carbon density was calculated based on-organic carbon content and bulk density and is expressed in Mg C (million grams of carbon) per ha to a depth of 25 cm.

### Stand biomass and carbon accounting

Forest tree inventory data were used to estimate stand volume and to calculate biomass carbon density. We used the bivariate tree volume equations published in the Common Forest Tree Volume Tables for Forest Inventory in Guangdong Province [15]. An appropriate equation was used to compute individual tree volume according to species group (Table 1). Forest stand volume was obtained by summing the individual tree volumes in a community.

**Table 1:** Bivariate tree volume equations for different species groups.

Species group	Bivariate tree volume equations
Softwood conifers	*V* = 0.0000798524 × *D* ^1.74220^ × *H* ^1.01198^
Soft hardwood broadleaved trees	*V* = 0.0000674286 × *D* ^1.87657^ × *H* ^0.92888^
Hard hardwood broadleaved trees	*V* = 0.0000601228 × *D* ^1.87550^ × *H* ^0.98496^

*V* is the volume of individual standing trees (m^3^), *D* is DBH (cm), and *H* is tree height (m).

Forest biomass was estimated using a volume-biomass model [16]. Previous research has clearly demonstrated a significant linear relationship between forest stand volume and biomass [17]. We estimated forest stand biomass at the community level according to the biomass-volume equation for evergreen broadleaved forests established by Xu et al. [18]:

Y = 0.9501 V + 17.5941

where *Y* is forest stand biomass, and *V* is forest stand volume. Individual tree biomass was back-calculated in proportion to its volume as a fraction of stand biomass.

Dry forest biomass comprises of approximately 50% carbon [19], therefore, we used a factor of 0.5 to convert forest stand biomass to carbon stock. Forest biomass carbon density is the amount of biomass carbon stock per unit area and is expressed in Mg/ha.

### Statistical analysis

The forest was further divided into groups of sample units using two-way indicator species analysis (TWINSPAN), a common technique for community classification [20]. In TWINSPAN setup, we used a user-defined pseudospecies cut level to obtain four arbitrary groups. The resulting TWINSPAN groups, which were considered communities, were used in subsequent analyses. Variations in species composition were assessed using multi-response permutation procedures (MRPP), a distribution-free and nonparametric statistical method that has been widely applied in ecological research for testing differences among two or more groups in a data matrix [21]. The importance value (IV) was calculated as the sum of relative abundance, relative frequency, and relative prominence (basal area) divided by three and was used as a criterion to determine the dominant species. To categorize the size class, which is commonly regarded as a surrogate for age class, three DBH classes were established: small (1 cm ≤ DBH < 7.5 cm), medium (7.5 cm ≤ DBH < 22.5 cm), and large (DBH ≥ 22.5 cm). The differences in mean DBH and tree height across various communities were evaluated using a one-way analysis of variance (ANOVA), followed by the least significant difference (LSD) test for multiple comparison if the ANOVA revealed an overall significant difference. TWINSPAN, MRPP, and species IV calculation were carried out using PC-ORD 6.08, while ANOVA and multiple comparisons were performed using STATISTICA version 8.0.

## Results

### Species composition and stand structure

A total of 18,025 trees were recorded within the sampling plot, and these belonged to 93 species in 64 genera and 39 families. Although different communities, as delimited by TWINSPAN, shared some common dominant species, the structural parameters of DBH and tree height were significantly different across the communities (Table 2). Significant differences in species composition across the communities were also revealed by MRPP (Table 3).

**Table 2:** Species composition, stand structure, and the dominant species across TWINSPAN groups.

Attribute	TWINSPAN groups			
	1	2	3	4
No. of quadrats	23	38	20	19
No. of species	48	67	66	71
Tree density (stems/ha)	3,765	4,754	4,420	4,999
DBH (cm)*	8.15 ± 0.14 a	6.59 ± 0.09 b	6.57 ± 0.12 b	5.19 ± 0.09 c
Tree height (m)*	8.45 ± 0.10 a	7.38 ± 0.06 b	7.79 ± 0.10 c	6.55 ± 0.08 d
Standing volume (m^3^)	307.01	430.72	227.54	126.71
Dominant species	*Castanopsis carlesii; Schima superba*; *Itea chinensis*; *Castanopsis fargesii; Machilus velutina*	*Castanopsis carlesii*; *Schima superba*; *Itea chinensis*; *Cunninghamia lanceolata*; *Litsea rotundifolia* var. *oblongifolia*;	*Castanopsis carlesii*; *Cratoxylon ligustrinum*; *Litsea rotundifolia* var. *oblongifolia*; *Camellia oleifera*; *Adinandra millittii*; *Itea chinensis*	*Cratoxylon ligustrinum*; *Camellia oleifera*; *Schima superba*; *Ilex pubescens*; *Photinia prunifolia*

*Differences in DBH and tree height across TWINSPAN groups were tested using one-way ANOVA followed by an LSD *post hoc* test of significance. Significant differences at P < 0.05 are indicated by different lowercase letters.

**Table 3:** MRPP for species composition among different TWINSPAN groups.

Group	Observed	Expected	Variance	Skewness	A	T	P
Overall comparison	49.10	61.61	0.28	-1.31	0.20	-23.44	< 0.001
Pairwise comparison							
4 *vs*. 1					0.30	-21.65	< 0.0001
4 *vs*. 2					0.24	-24.91	< 0.0001
4 *vs*. 3					0.08	-9.0	< 0.0001
3 *vs*. 1					0.14	-13.05	< 0.0001
3 *vs*. 2					0.08	-10.48	< 0.0001
2 *vs*. 1					0.02	-3.13	< 0.05

A, a descriptor of within group homogeneity, compared to the random expectation; T, a statistic describes the separation between the groups; P, the actual P-value from the significance test of homogeneity.

### Community-level variation in biomass and soil carbon densities

**Fig 1:**
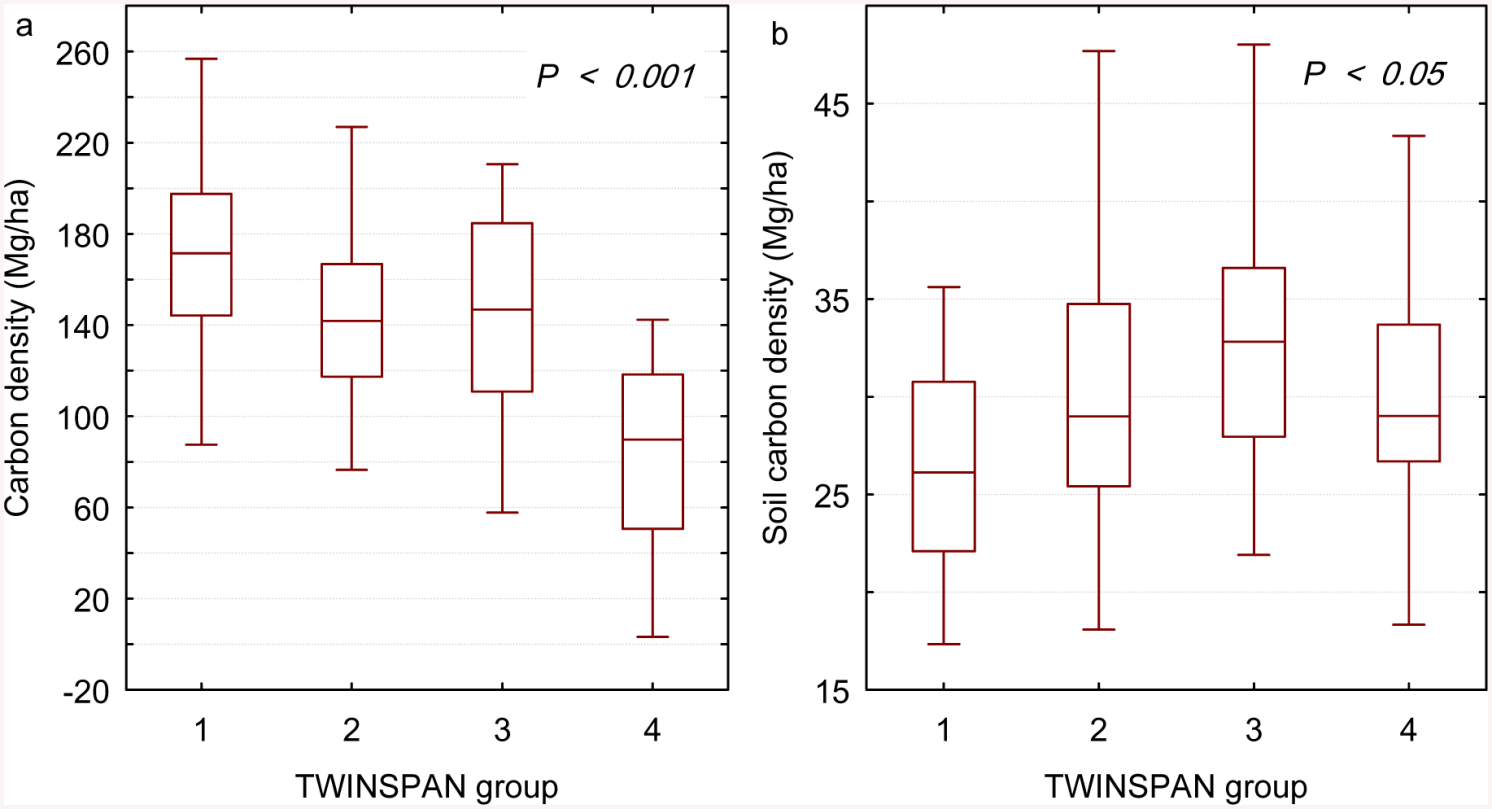
Forest biomass carbon density (a) and soil organic carbon density (b) across TWINSPAN groups. The horizontal line in each box is the median, the box endpoints indicate the 25th and 75th percentile values, and the whiskers represent the non-outlier range.

The average biomass carbon density of the trees in the sample plot was 136.34 Mg/ha. Significant variations were found in biomass carbon density across the four TWINSPAN groups (Fig 1a; Kruskal-Wallis test, P < 0.001). Multiple comparisons revealed that the carbon density significantly differed (P < 0.05) between groups except between TWINSPAN groups 2 and 3. Topsoil organic carbon density also differed significantly among groups (Fig 1b; Kruskal-Wallis test, P < 0.05) but in a different way than biomass carbon density (Fig 1). Group 4 had the lowest biomass carbon density, possibly due to the relative predominance of species with small-sized individuals, such as *Cratoxylon ligustrinum*, *Camellia oleifera*, and *Ilex pubescens* in this group.

### Species-level variation in biomass carbon density

The dominant species in the sample plot were tree species that are common and indigenous to the southern subtropical area (Table 4). The 10 most dominant species combined were responsible for 89.7% of the total biomass carbon stock of the forest stand, and the two most dominant species as sorted by importance value (IV) had carbon stocks of 259.56 Mg and 101.20 Mg, accounting for 46.86% and 18.27% of the total biomass carbon storage, respectively (Table 4). For these dominant species, a lower IV was generally associated with a smaller carbon stock, while the basal area was a direct indicator of carbon stock change.

**Table 4:** The carbon stock of the dominant species and of other species in the forest stand.

Species	Importance value (%)	Basal area (dm^2^)	Standing volume (m^3^)	Carbon stock (Mg)	Carbon density (Mg/ha)	% of the total carbon stock
*Castanopsis carlesii*	20.57	5,881.85	511.67	259.56	64.89	46.86
*Schima superba*	12.81	2,533.44	199.50	101.20	25.30	18.27
*Castanopsis fargesii*	5.25	1,354.00	117.39	59.55	14.89	10.75
*Itea chinensis*	3.43	103.94	4.02	2.04	0.51	0.37
*Cunninghamia lanceolata*	3.08	482.16	32.48	16.48	4.12	2.97
*Litsea rotundifolia* var. *oblongifolia*	2.86	41.03	1.50	0.76	0.19	0.14
*Cinnamomum porrectum*	2.83	413.52	30.82	15.63	3.91	2.82
*Castanopsis hystrix*	2.65	565.67	49.16	24.94	6.23	4.50
*Engelhardtia roxburghiana*	2.54	375.47	28.46	14.44	3.61	2.61
*Photinia prunifolia*	2.18	98.81	4.54	2.30	0.58	0.42
Other species in total (83 species)	41.8	1,757.00	112.43	37.03	9.26	10.30

### Differences in biomass carbon stock across DBH classes

**Fig 2:**
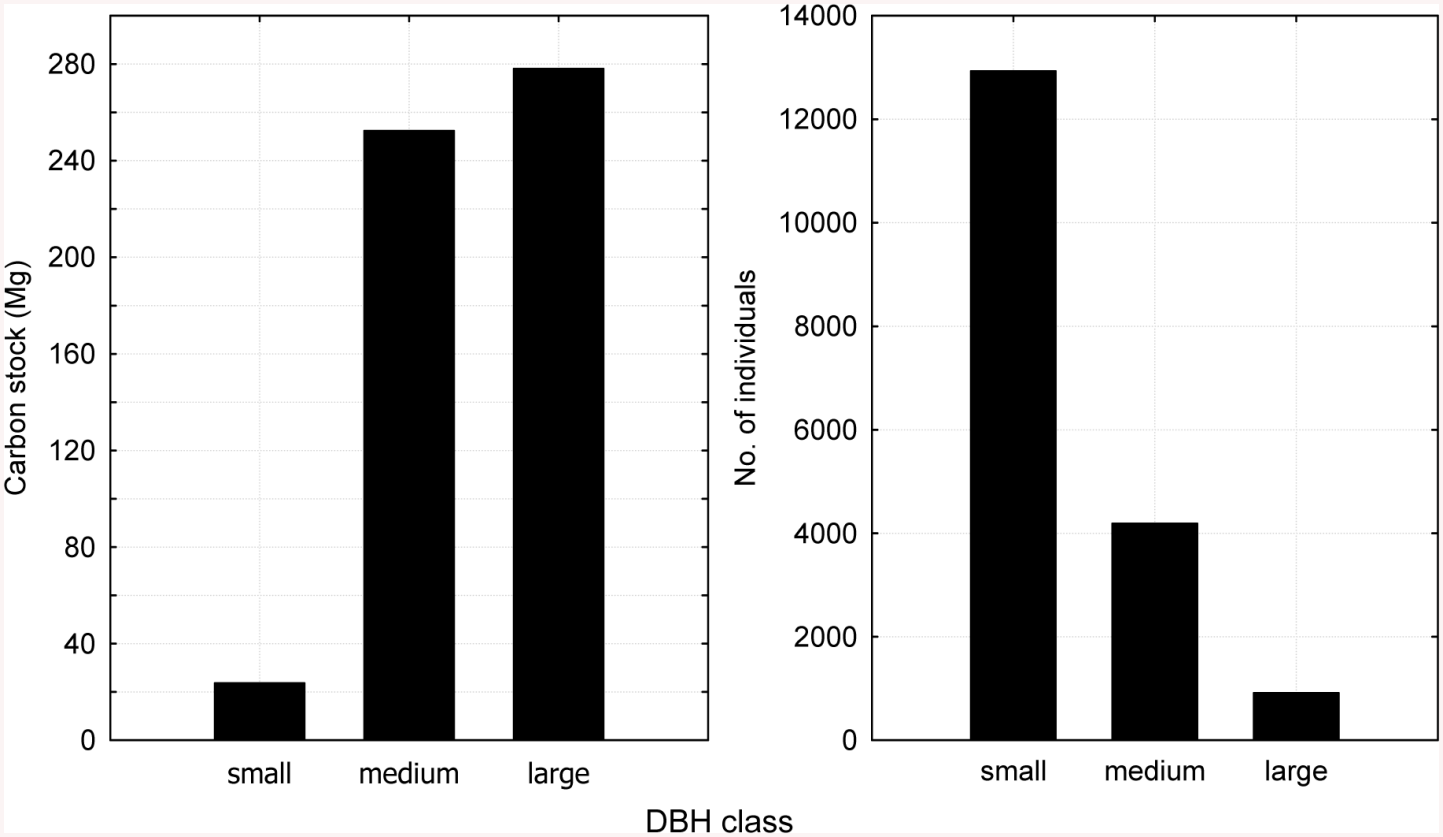
Forest biomass carbon stock and the number of individuals in different DBH classes.

**Fig 3:**
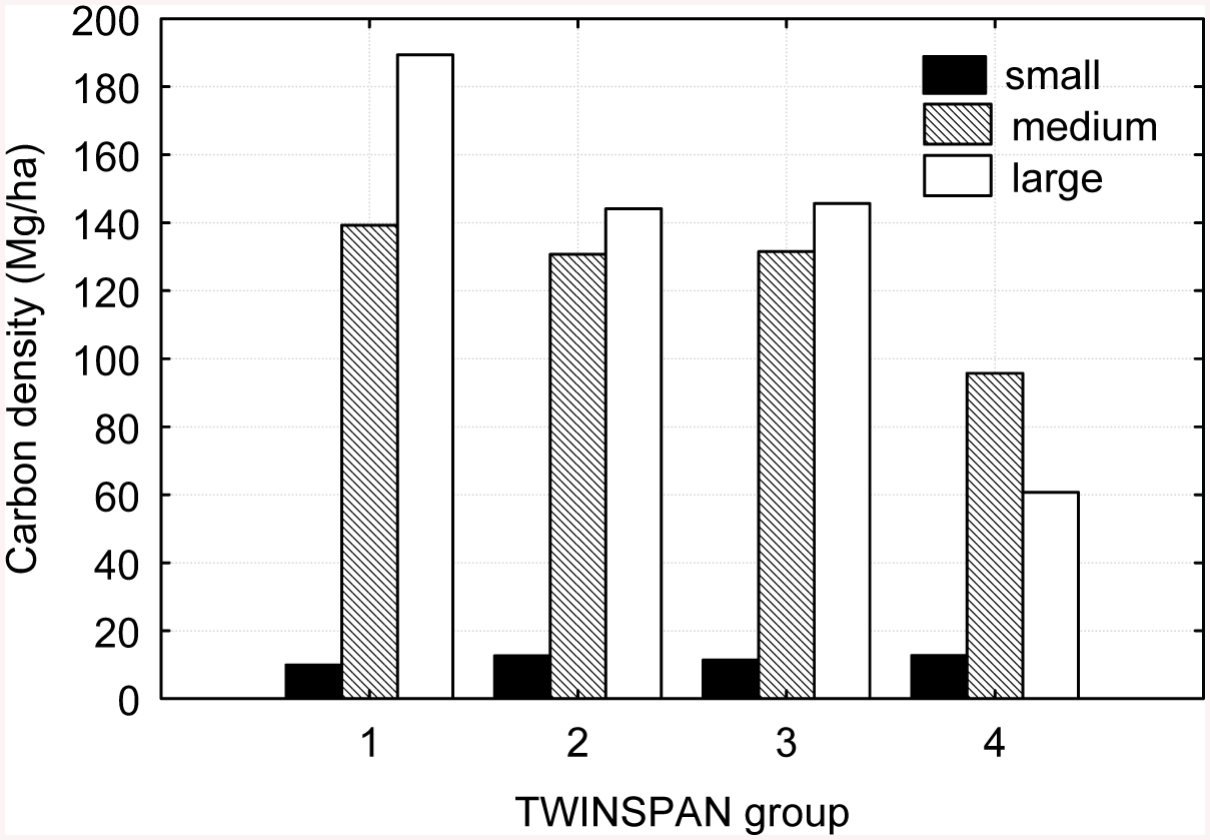
Forest biomass carbon density of various DBH classes across TWINSPAN groups.

Biomass carbon stock significantly differed (P < 0.05) among diameter classes of standing trees. A sharp contrast was found between carbon stock and total tree abundance across the DBH classes. With only 912 individuals (5.06% of total abundance), trees of the large-diameter class contributed 50.19% to the total biomass carbon stock; in contrast, with 12,928 individuals (71.72% of total abundance), the small-diameter class contributed only 23.60 Mg to the total biomass carbon stock, accounting for 4.26% of the total. Standing trees of the medium- and large-diameter classes together represented only 28.27% of the total number of individuals but 95.74% of the total biomass carbon stock (Fig 2). A similar trend was evident across the TWINSPAN groups (Fig 3).

## Discussion

The average tree biomass carbon density in the sample plot was 136.34 Mg/ha, which is higher than the national average biomass carbon density of all forests (41.0 Mg/ha) [22] and higher than the national average biomass carbon density of evergreen broadleaved forests in China (73.68 Mg/ha) [22]. Both biomass carbon density and topsoil organic carbon density exhibited significant spatial variation. Species composition and community structure had significant impacts on biomass carbon density. The dominant species in the entire forest stand were the major contributors to forest biomass carbon accumulation. The carbon stock of the 10 most dominant species accounted for 89.7% of the total biomass carbon storage, and the two most dominant species alone, *Castanopsis carlesii* and *Schima superba*, contributed 65.13% to the total biomass carbon stock, indicating that the bulk of the carbon pool is determined by a small number of species in a subtropical evergreen forest with high species dominance.

An inverse association was found between tree abundance and its contribution to biomass carbon stock. Standing trees in the medium- and large-diameter classes were the primary contributors to stand biomass carbon storage. Although they represented only 28.28% of the total number of trees, trees in the medium- and large-DBH classes contributed 95.74% to the total biomass carbon stock, whereas the majority of trees (12,928) were in the small-diameter class and contributed only 4.26% to the total biomass carbon stock. This result also indicated that, although the majority of current forest biomass carbon storage was contributed by medium- and large-sized trees, with a large number of seedlings and saplings, the forest stand had a high capacity for forest regeneration and thus a great potential for continuous carbon accumulation.

A high proportion of the soil organic carbon stock is generally concentrated in the upper soil layers [23]. In the southern subtropical forest area of China, the topsoil organic carbon stock is especially high and more closely related to vegetation patterns than in other areas [24]. In our study, topsoil organic carbon density varied significantly across TWINSPAN groups (Kruskal-Wallis test, P < 0.05), but it changed in a different way than biomass carbon density. This observation suggests that the mechanisms of and relationships between biomass carbon and soil organic carbon accumulation may be very complex [25, 26]. The accumulation of biomass carbon results from tree growth; in a natural forest stand, however, tree growth may deplete soil fertility, which is positively associated with soil organic carbon accumulation [27].

In this study, we demonstrate the impact of species composition and community structure on forest biomass carbon and soil carbon. Current forest biomass carbon accounting protocols fail to include these attributes. The volume-biomass model we used assumes that all the tree species of the same species group are homogeneous in wood density; however, species-specific parameters including wood density and biomass carbon ratio should be investigated to make local parameters available for future accurate forest carbon estimation.

## Conclusions

Species composition and community structure had significant impacts on both the biomass carbon density and soil organic carbon density of the forest stand. Our study has three practical implications for forest management and forest carbon accounting in both CDM and non-CDM forest projects. First, the heterogeneity in species composition and stand structure should be considered for accurate forest carbon accounting. Second, when measuring forest carbon accumulation per unit time, e.g., per year, researchers can resample only a few dominant species to save time and money while maintaining adequate accuracy. Third, in afforestation and forestry activities for carbon sequestration, species selection should be based on the dominant species from the local natural forest because they are more adaptable to the local climate and soil environment and are the major contributors to forest biomass carbon accumulation.

## Supporting Information

S1 Table Forest biomass carbon density and soil organic carbon density in TWINSPAN-delimited communities. (DOCX)

S2 Table Biomass carbon density and the number of individuals in different DBH classes in TWINSPAN-delimited communities. (DOCX)

S3 Table Checklist of species occurred in the sample plot and the structural attributes of each species. (DOCX)
