# Supplementary material for: Influence of Tree Species Composition and Community Structure on Carbon Density in a Subtropical Forest
Source: PLoS One. 2015 Aug 28;10(8):e0136984. doi: 10.1371/journal.pone.0136984 (PMC4552639; doi:10.1371/journal.pone.0136984)
Supplement: S1 Table — (DOCX) [file pone.0136984.s001.docx]

S1 Table. Forest biomass carbon density and soil organic carbon density in TWINSPAN-delimited communities.

| Community | Standing volume (m^3^) | Biomass (Mg) | Carbon storage (Mg) | Carbon density (Mg/ha) | Organic carbon content (g/kg) | Soil carbon storage (Mg) | Soil carbon density (Mg/ha) |
| --- | --- | --- | --- | --- | --- | --- | --- |
| 1 | 307.01 | 309.28 | 154.64 | 168.09 | 16.89 | 24.96 | 27.13 |
| 2 | 430.72 | 426.82 | 213.41 | 140.40 | 17.9 | 45.23 | 29.76 |
| 3 | 227.54 | 233.78 | 116.89 | 146.11 | 16.88 | 26.40 | 33.00 |
| 4 | 126.72 | 137.98 | 68.99 | 90.78 | 15.59 | 22.96 | 30.21 |
