# Supplementary material for: Influence of Tree Species Composition and Community Structure on Carbon Density in a Subtropical Forest
Source: PLoS One. 2015 Aug 28;10(8):e0136984. doi: 10.1371/journal.pone.0136984 (PMC4552639; doi:10.1371/journal.pone.0136984)
Supplement: S2 Table — (DOCX) [file pone.0136984.s002.docx]

S2 Table. Biomass carbon density and the number of individuals in different DBH classes in TWINSPAN-delimited communities.

| Community | DBH class | No. of individuals | Carbon density (Mg/ha) |
| --- | --- | --- | --- |
| 1 | small | 2205 | 9.94 |
|  | medium | 972 | 139.23 |
|  | large | 287 | 189.39 |
| 2 | small | 5199 | 12.69 |
|  | medium | 1665 | 130.66 |
|  | large | 363 | 144.14 |
| 3 | small | 2543 | 11.39 |
|  | medium | 816 | 131.51 |
|  | large | 177 | 145.65 |
| 4 | small | 2981 | 12.71 |
|  | medium | 732 | 95.72 |
|  | large | 85 | 60.71 |
