# Supplementary material for: Influence of Tree Species Composition and Community Structure on Carbon Density in a Subtropical Forest
Source: PLoS One. 2015 Aug 28;10(8):e0136984. doi: 10.1371/journal.pone.0136984 (PMC4552639; doi:10.1371/journal.pone.0136984)
Supplement: S3 Table — (DOCX) [file pone.0136984.s003.docx]

S3 Table. Checklist of species occurred in the sample plot and the structural attributes of each species.

| Species | No. of trees | Averaged DBH (cm) | Averaged Height (m) | Basal area (dm^2^) | Relative Frequency (%) | Relative Density (%) | Relative Dominance (%) | Importance Value (%) |
| --- | --- | --- | --- | --- | --- | --- | --- | --- |
| *Adenanthera pavonina* | 2 | 4.6 | 6.4 | 0.37 | 0.08 | 0.01 | 0 | 0.03 |
| *Adinandra millittii* | 478 | 2.6 | 4.5 | 34.96 | 2.5 | 2.39 | 0.26 | 1.72 |
| *Aidia canthioides* | 60 | 3 | 4.4 | 7.93 | 0.95 | 0.3 | 0.06 | 0.44 |
| *Alangium chinense* | 34 | 13 | 11.6 | 65.6 | 0.68 | 0.17 | 0.48 | 0.44 |
| *Albizia kalkora* | 3 | 5.3 | 7.2 | 0.9 | 0.12 | 0.02 | 0.01 | 0.05 |
| *Albizia macrophylla* | 2 | 6.9 | 12.5 | 0.95 | 0.04 | 0.01 | 0.01 | 0.02 |
| *Alniphyllum fortunei* | 3 | 3.9 | 9.3 | 0.38 | 0.08 | 0.02 | 0 | 0.03 |
| *Antidesma japonicum* | 19 | 1.7 | 3 | 0.51 | 0.2 | 0.1 | 0 | 0.1 |
| *Artocarpus hypargyraeus* | 3 | 5 | 6.9 | 1.02 | 0.08 | 0.02 | 0.01 | 0.03 |
| *Beilschmiedia tsangii* | 267 | 4.4 | 5.6 | 75.62 | 2.15 | 1.34 | 0.55 | 1.35 |
| *Bridelia fordii* | 19 | 2.9 | 4 | 1.51 | 0.2 | 0.1 | 0.01 | 0.1 |
| *Camellia oleifera* | 666 | 2.1 | 3.1 | 27.47 | 1.83 | 3.33 | 0.2 | 1.79 |
| *Castanopsis carlesii* | 2991 | 12.2 | 11.7 | 5881.85 | 3.78 | 14.96 | 42.99 | 20.57 |
| *Castanopsis eyrei* | 10 | 17.1 | 14 | 32.44 | 0.12 | 0.05 | 0.24 | 0.14 |
| *Castanopsis fargesii* | 508 | 14.8 | 12.6 | 1354 | 3.3 | 2.54 | 9.9 | 5.25 |
| *Castanopsis hystrix* | 300 | 12.3 | 11.8 | 565.67 | 2.31 | 1.5 | 4.13 | 2.65 |
| *Casearia villilimba* | 49 | 3.1 | 4.6 | 5.33 | 0.95 | 0.25 | 0.04 | 0.41 |
| *Celtis biondii* | 2 | 1.6 | 4.5 | 0.04 | 0.04 | 0.01 | 0 | 0.02 |
| *Celtis tetrandra* ssp. *sinensis* | 3 | 7.5 | 5.5 | 1.32 | 0.08 | 0.02 | 0.01 | 0.03 |
| *Cerasus campanulata* | 2 | 4.9 | 5 | 0.56 | 0.08 | 0.01 | 0 | 0.03 |
| *Choerospondias axillaris* | 83 | 15.7 | 13.9 | 215.42 | 1.31 | 0.42 | 1.57 | 1.1 |
| *Cinnamomum porrectum* | 416 | 9.5 | 10.7 | 413.52 | 3.38 | 2.08 | 3.02 | 2.83 |
| *Cratoxylum ligustrinum* | 785 | 3 | 4.7 | 78.25 | 1.11 | 3.93 | 0.57 | 1.87 |
| *Cryptocarya chinensis* | 33 | 5.7 | 6 | 12.6 | 0.6 | 0.17 | 0.09 | 0.28 |
| *Cunninghamia lanceolata* | 619 | 8.5 | 8.7 | 482.16 | 2.62 | 3.1 | 3.52 | 3.08 |
| *Dalbergia balansae* | 2 | 2.3 | 4.6 | 0.11 | 0.08 | 0.01 | 0 | 0.03 |
| *Daphniphyllum calycinum* | 17 | 3.8 | 4.4 | 2.57 | 0.32 | 0.09 | 0.02 | 0.14 |
| *Daphniphyllum macropodum* | 1 | 12.5 | 15.5 | 1.23 | 0.04 | 0.01 | 0.01 | 0.02 |
| *Daphniphyllum oldhamii* | 37 | 4.1 | 5.3 | 8.96 | 0.76 | 0.19 | 0.07 | 0.34 |
| *Diospyros eriantha* | 1 | 2.4 | 5 | 0.05 | 0.04 | 0.01 | 0 | 0.02 |
| *Diospyros kaki* | 50 | 4.8 | 6.3 | 16.39 | 0.68 | 0.25 | 0.12 | 0.35 |
| *Diospyros morrisiana* | 326 | 3.5 | 5.3 | 43.14 | 3.1 | 1.63 | 0.32 | 1.68 |
| *Ehretia longiflora* | 8 | 5.7 | 7 | 3.45 | 0.16 | 0.04 | 0.03 | 0.07 |
| *Elaeocarpus chinensis* | 308 | 3.4 | 4.9 | 37.04 | 2.82 | 1.54 | 0.27 | 1.54 |
| *Elaeocarpus japonicus* | 1 | 3.4 | 8.3 | 0.09 | 0.04 | 0.01 | 0 | 0.02 |
| *Elaeocarpus sylvestris* | 21 | 6.3 | 7.1 | 11.81 | 0.64 | 0.11 | 0.09 | 0.28 |
| *Endospermum chinense* | 2 | 1.4 | 3.5 | 0.03 | 0.04 | 0.01 | 0 | 0.02 |
| *Engelhardtia roxburghiana* | 432 | 8.2 | 9.5 | 375.47 | 2.7 | 2.16 | 2.74 | 2.54 |
| *Eurya distichophylla* | 4 | 1.4 | 2.5 | 0.06 | 0.12 | 0.02 | 0 | 0.05 |
| *Eurya japonica* | 1 | 3 | 3.3 | 0.07 | 0.04 | 0.01 | 0 | 0.02 |
| *Eurya macartneyi* | 309 | 2.2 | 3.3 | 15.96 | 2.47 | 1.55 | 0.12 | 1.38 |
| *Evodia lepta* | 15 | 2.6 | 3.8 | 1.27 | 0.28 | 0.08 | 0.01 | 0.12 |
| *Evodia meliaefolia* | 2 | 24 | 14 | 9.11 | 0.04 | 0.01 | 0.07 | 0.04 |
| *Ficus fistulosa* | 8 | 3.3 | 3.6 | 0.8 | 0.08 | 0.04 | 0.01 | 0.04 |
| *Ficus superba* var. *japonica* | 26 | 9.4 | 7.2 | 32.41 | 0.24 | 0.13 | 0.24 | 0.2 |
| *Ficus variolosa* | 181 | 2.6 | 4.1 | 14.82 | 2.43 | 0.91 | 0.11 | 1.15 |
| *Garcinia multiflora* | 51 | 3.6 | 3.8 | 8.1 | 0.4 | 0.26 | 0.06 | 0.24 |
| *Garcinia oblongifolia* | 3 | 2.6 | 3.7 | 0.2 | 0.08 | 0.02 | 0 | 0.03 |
| *Homalium cochinchinense* | 60 | 2.1 | 3.8 | 2.79 | 0.91 | 0.3 | 0.02 | 0.41 |
| *Hovenia acerba* | 1 | 9.3 | 17.6 | 0.68 | 0.04 | 0.01 | 0 | 0.02 |
| *Ilex pubescens* | 577 | 1.4 | 2.7 | 13.62 | 3.3 | 2.89 | 0.1 | 2.1 |
| *Ilex rotunda* | 4 | 5.8 | 8 | 1.23 | 0.08 | 0.02 | 0.01 | 0.04 |
| *Itea chinensis* | 1205 | 2.9 | 4.5 | 103.94 | 3.5 | 6.03 | 0.76 | 3.43 |
| *Ixonanthes chinensis* | 24 | 7.6 | 8.9 | 15.49 | 0.28 | 0.12 | 0.11 | 0.17 |
| *Lindera communis* | 12 | 5.6 | 5.8 | 5.97 | 0.24 | 0.06 | 0.04 | 0.11 |
| *Liquidambar formosana* | 72 | 8.9 | 9.4 | 61.09 | 0.68 | 0.36 | 0.45 | 0.49 |
| *Litsea cubeba* | 12 | 2.9 | 5.3 | 1.32 | 0.12 | 0.06 | 0.01 | 0.06 |
| *Litsea rotundifolia* var*. oblongifolia* | 927 | 2.1 | 4.6 | 41.03 | 3.66 | 4.64 | 0.3 | 2.86 |
| *Machilus breviflora* | 2 | 10.9 | 16 | 1.94 | 0.08 | 0.01 | 0.01 | 0.03 |
| *Machilus chinensis* | 166 | 5.8 | 7.1 | 81.98 | 2.47 | 0.83 | 0.6 | 1.3 |
| *Machilus pauhoi* | 2 | 5.8 | 8 | 0.53 | 0.04 | 0.01 | 0 | 0.02 |
| *Machilus velutina* | 582 | 1.6 | 2.8 | 14.89 | 3.46 | 2.91 | 0.11 | 2.16 |
| *Mallotus philippinensis* | 6 | 5.9 | 4.3 | 3.6 | 0.24 | 0.03 | 0.03 | 0.1 |
| *Myrica rubra* | 32 | 7.7 | 8.3 | 20.61 | 0.52 | 0.16 | 0.15 | 0.28 |
| *Nyssa sinensis* | 2 | 10.3 | 10.8 | 1.69 | 0.08 | 0.01 | 0.01 | 0.03 |
| *Paulownia fortunei* | 3 | 14 | 8.3 | 7.57 | 0.08 | 0.02 | 0.06 | 0.05 |
| *Photinia beauverdiana* | 9 | 10.8 | 10.9 | 9.56 | 0.24 | 0.05 | 0.07 | 0.12 |
| *Photinia prunifolia* | 566 | 3.6 | 5.3 | 98.81 | 2.98 | 2.83 | 0.72 | 2.18 |
| *Pinus massoniana* | 33 | 20.1 | 15.4 | 122.9 | 0.4 | 0.17 | 0.9 | 0.49 |
| *Pithecellobium clypearia* | 25 | 11 | 11.5 | 36.95 | 0.64 | 0.13 | 0.27 | 0.34 |
| *Pithecellobium lucidum* | 3 | 2.3 | 4.2 | 0.14 | 0.08 | 0.02 | 0 | 0.03 |
| *Pterospermum heterophyllum* | 282 | 5.8 | 7.3 | 128.94 | 1.11 | 1.41 | 0.94 | 1.16 |
| *Pyrus calleryana* | 48 | 3.9 | 5.4 | 9.01 | 0.56 | 0.24 | 0.07 | 0.29 |
| *Pyrus pyrifolia* | 2 | 11.3 | 13 | 2.02 | 0.04 | 0.01 | 0.01 | 0.02 |
| *Reevesia thyrsoidea* | 8 | 5.3 | 7.8 | 2.98 | 0.12 | 0.04 | 0.02 | 0.06 |
| *Rhodomyrtus tomentosa* | 52 | 1.8 | 3 | 1.59 | 0.44 | 0.26 | 0.01 | 0.24 |
| *Rhus chinensis* | 4 | 1.7 | 3.4 | 0.1 | 0.04 | 0.02 | 0 | 0.02 |
| *Sapium discolor* | 215 | 10.3 | 12.9 | 234.21 | 2.54 | 1.08 | 1.71 | 1.78 |
| *Sapindus saponaria* | 1 | 19.8 | 11 | 3.08 | 0.04 | 0.01 | 0.02 | 0.02 |
| *Schefflera octophylla* | 177 | 6 | 5.6 | 81.28 | 2.43 | 0.89 | 0.59 | 1.3 |
| *Schima superba* | 3200 | 7.8 | 9 | 2533.44 | 3.9 | 16.01 | 18.52 | 12.81 |
| *Styrax odoratissima* | 218 | 3.5 | 5.4 | 26.69 | 2.07 | 1.09 | 0.2 | 1.12 |
| *Styrax suberifolia* | 122 | 6.3 | 8.2 | 54.23 | 0.95 | 0.61 | 0.4 | 0.65 |
| *Symplocos lancifolia* | 27 | 4.1 | 5 | 5.1 | 0.44 | 0.14 | 0.04 | 0.2 |
| *Syzygium buxifolium* | 11 | 1.4 | 2.2 | 0.17 | 0.16 | 0.06 | 0 | 0.07 |
| *Syzygium grijsii* | 49 | 1.5 | 2.5 | 0.94 | 0.6 | 0.25 | 0.01 | 0.28 |
| *Syzygium rehderianum* | 4 | 2.1 | 5 | 0.17 | 0.08 | 0.02 | 0 | 0.03 |
| *Toxicodendron succedaneum* | 82 | 3.4 | 6 | 11.74 | 1.23 | 0.41 | 0.09 | 0.58 |
| *Tutcheria championii* | 3 | 14.3 | 13.2 | 4.9 | 0.04 | 0.02 | 0.04 | 0.03 |
| *Vernicia montana* | 4 | 18 | 15 | 10.15 | 0.16 | 0.02 | 0.07 | 0.08 |
| *Vitex negundo* | 10 | 2.2 | 4.8 | 0.42 | 0.24 | 0.06 | 0 | 0.1 |
| *Vitex quinata* | 16 | 4 | 5.4 | 3.79 | 0.32 | 0.08 | 0.03 | 0.14 |
| *Zanthoxylum myriacanthum* | 2 | 2.4 | 4.2 | 0.09 | 0.04 | 0.01 | 0 | 0.02 |
